# Mechanistic Understanding of D-Glucaric Acid to Support Liver Detoxification Essential to Muscle Health Using a Computational Systems Biology Approach

**DOI:** 10.3390/nu15030733

**Published:** 2023-02-01

**Authors:** V. A. Shiva Ayyadurai, Prabhakar Deonikar, Christine Fields

**Affiliations:** 1 Systems Biology Group, CytoSolve Research Division, CytoSolve, Inc., Cambridge, MA 02138, USA; 2 Applied Food Sciences Inc., 8708 South Congress Suite 290, Austin, TX 78745, USA

**Keywords:** liver toxicity, systems biology, D-glucaric acid, muscle soreness, mathematical modeling, CytoSolve

## Abstract

Liver and muscle health are intimately connected. Nutritional strategies that support liver detoxification are beneficial to muscle recovery. Computational–in silico–molecular systems’ biology analysis of supplementation of calcium and potassium glucarate salts and their metabolite D-glucaric acid (GA) reveals their positive effect on mitigation of liver detoxification via four specific molecular pathways: (1) ROS production, (2) deconjugation, (3) apoptosis of hepatocytes, and (4) β-glucuronidase synthesis. GA improves liver detoxification by downregulating hepatocyte apoptosis, reducing glucuronide deconjugates levels, reducing ROS production, and inhibiting β-Glucuronidase enzyme that reduces re-absorption of toxins in hepatocytes. Results from this in silico study provide an integrative molecular mechanistic systems explanation for the mitigation of liver toxicity by GA.

## 1. Introduction

Muscle soreness and liver health are interconnected systems [1,2,3]. Muscle soreness has been shown to upregulate biomarkers of liver damage and liver toxicity [4,5,6,7], and liver detoxification has been shown to support muscle recovery [8]. Dietary supplementation has been shown to ameliorate muscle and liver damage [6,9]; however, the mechanistic reasons of such benefits are not yet fully understood. 

D-glucaric acid (GA) is a natural and non-toxic compound that can improve the metabolic cleansing process and aid biotransformation [10]. Dietary calcium or potassium glucarate salts have been shown to increase the level of GA in the serum [11]. GA and its derivative D-saccharic acid-1,4-lactone (DSL) have been shown to have hepatoprotective [12,13], anti-inflammatory, cholesterol lowering, anti-oxidant [12], and anti-carcinogenic [11] effects. In this study, we focus on understanding the underlying mechanisms of action of GA on liver damage and liver detoxification. 

Detoxification is an important cellular task that involves mobilization, modification, and excretion of exogenous and endogenous toxicants [14,15]. Common toxicants include heavy metals, persistent organic pollutants, electromagnetic radiation, stress, fat metabolites, alcohol metabolite, pharmaceutical and recreational drugs, and bacterial endotoxins [16,17]. Liver is the first filter organ between the gastrointestinal tracts and the rest of the body, providing critical detoxification processes [14]. The majority of the detoxification and biotransformation processes occur in the liver [18]. The process of detoxification in the liver involves multiple steps in the biotransformation of primarily non-polar, lipid-soluble toxicants into polar, water-soluble, and excretable derivatives, which are classified as Phase-I and Phase-II detoxification pathways [14]. Any dysfunction in detoxification processes leads to an accumulation of toxins and initiation of early morbidity and mortality [17].

A growing body of evidence has shown that foods rich with glucarate salts, such as apple, grapefruit, alfalfa sprouts, etc., may upregulate or favorably balance metabolic pathways to assist liver detoxification [19,20,21]. Such findings suggest that dietary supplementation of glucarate salts may benefit liver detoxification processes.

While there is substantial empirical evidence suggesting the role of the metabolites of glucarate salts in aiding liver detoxification processes, the mechanistic explanation of how these metabolites exert such beneficial effect is not clearly understood. In this study, the research aim is to understand the effect of GA on the molecular pathways of liver detoxification processes. Such understanding demands the need to uncover complex molecular systems that conventional in vitro and in vivo methods find difficult to elicit. 

Emerging modern bioinformatics and computational systems biology methodologies, performed in silico-meaning on the computer, provide the opportunity to explore such complex systems. Once such platform is CytoSolve^®^ (version 5.204) which is a well-established computational systems biology framework of technology and processes that provide the capability to derive molecular mechanisms of action, to create quantitative and predictive models of those mechanisms, and to employ the resultant models to simulate complex biomolecular phenomena [22,23,24,25,26,27]. The study herein employs CytoSolve, a proven computational systems biology approach to: (1) identify potential molecular mechanisms involved in liver detoxification affected by GA; and (2) quantitatively predict the effect of GA in aiding liver detoxification. Previous work has demonstrated the viability of using such a computational systems biology approach to model complex biomolecular phenomena [23,26,27,28,29,30,31].

## 2. Materials and Methods

The methodology used to identify the mechanisms of action of liver detoxification and to quantitatively predict the effects of GA on such mechanisms is described in this section. The CytoSolve^®^ computational systems biology platform was employed in this process. The protocol for setting up and using CytoSolve^®^ is explained in detailed by Ayyadurai and Deonikar, 2022 [31], and briefly described in Supplementary File S1. 

### 2.1. Systematic Literature Review Process and Inclusion Criteria

The workflow for the identification, organization, and curation of the literature and the extraction of information from the literature was performed per the standardized CytoSolve^®^ protocol detailed in previous studies [29,30,31]. The specific list of Medical Subject Headings (MeSH) keywords is provided in File S3 in Supplementary Materials Section S3.1. 

**Figure 1:**
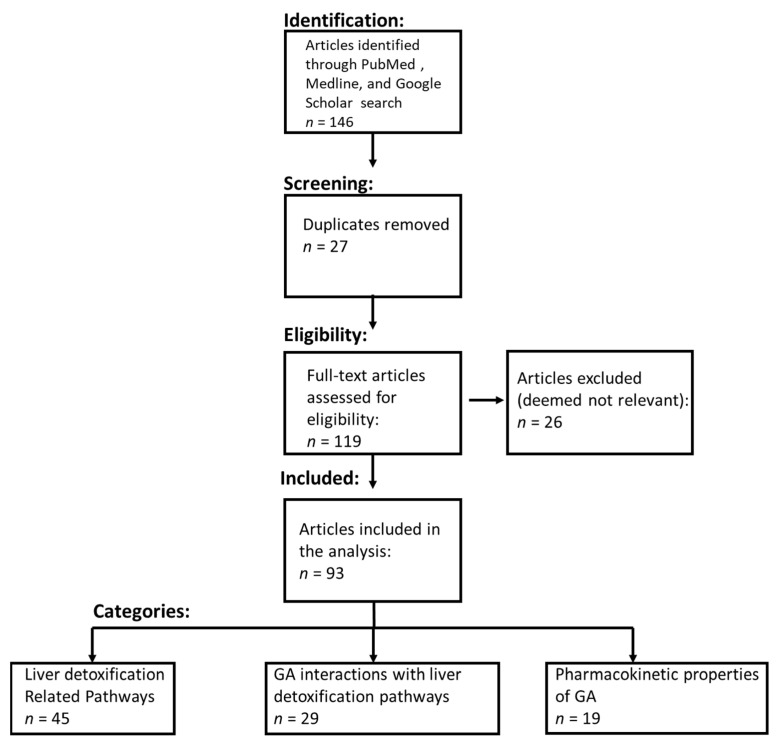
PRISMA flow diagram. The systematic literature review process included identifying the relevant literature from PubMed, Medline, and Google Scholar. The literature was then filtered to remove duplicate studies. The eligibility of articles for the comprehensive review was determined using the inclusion criteria detailed in the Materials and Methods section.

Using the keywords in File S3, the relevant retrieved articles are categorized and represented in Figure 1 and follow PRISMA guidelines [32]. 

### 2.2. CytoSolve in Silico Modeling Protocol

The identification and extraction of data related to reaction rate constants, biochemical reactions, and pharmacokinetic properties of GA with respect to the molecular pathways of liver detoxification were performed per the standardized CytoSolve^®^ protocol detailed in previous studies [29,30,31]. All biochemical reactions for each of the individual liver detoxification mathematical models, along with the kinetic parameters and the initial concentration of biochemical species, are listed in Supplementary File S2 in Tables S2.1–S2.4.

Molecular pathways of liver detoxification are converted into individual mathematical models using the biochemical reactions per the standardized CytoSolve^®^ protocol detailed in previous studies [29,30,31]. The individual mathematical models were integrated using the standardized CytoSolve^®^ protocol detailed in previous studies [29,30,31].

#### 2.2.1. Control Conditions

In this study, the control condition denotes the in silico experimental condition where supplementation of GA is set to zero. Using this control condition, all four models involved in liver detoxification were simulated to find the concentrations of their respective biomarkers—ROS, glucuronide deconjugate, cPARP, and β-glucuronidase—in the absence of GA supplementation. The values of these four biomarkers under control conditions were then compared with those obtained in the presence of GA supplementation to understand how GA affects these biomarkers.

For the ROS production model, the hepatocytes were assumed to be under oxidative hepatic injury condition, which increases the production ROS 10-fold [33] than the normal. Using this as an initial condition, the ROS production model was simulated over a period of two (2) days to predict the steady state control value of ROS to be 70 nM.

For the deconjugation/deglucuronidation model, the cell was assumed to be in an infectious state with elevated endotoxin levels of 0.1 nM [34]. Using this as an initial condition, the deconjugation model was simulated over a period of two days to predict the steady state control value of glucuronide deconjugates to be 0.0049 nM.

For hepatic apoptosis model, the cell was assumed to be in an infectious state with elevated pro-inflammatory cytokine TNF-α level of 0.0012 nM [35]. Using this as an initial condition, the hepatic apoptosis model was simulated over a period of two days to predict the steady state control value of cPARP to be 1217 nM.

For β-glucuronidase synthesis model, the cell was assumed to be in an infectious state with elevated endotoxin levels of 0.1 nM [34]. Using this as an initial condition, β-glucuronidase synthesis model was simulated over a period of two days to predict the steady state control value of β-glucuronidase levels to be 4.75 nM. 

#### 2.2.2. Computer Simulations to Study the Effect of GA on Integrated Model of Liver Detoxification

The integrated model of liver detoxification includes four major sub-systems: (1) hepatic apoptosis, (2) ROS production, (3) deconjugation/deglucuronidation, and (4) β-glucuronidase synthesis. The effect of GA was assessed by estimating the cellular concentration levels of biomarkers of these processes such as cPARP, reactive oxygen species, deconjugates, and β-glucuronidase, respectively, in the presence and absence of the GA. The integrated model of detoxification was simulated using the standardized CytoSolve^®^ protocol detailed in previous studies [29,30,31]. 

Input dosage levels of GA in simulation of all the models were based on its amounts present in some of the natural sources such as grapefruit. GA levels were found as low as 1.8 mg to as high as 120 mg in 100 mL of grapefruit juice of different varieties that are predominantly consumed [20]. In this study, we used GA concentrations of 1.8, 26, 52, and 120 mg/100 mL of grapefruit juice to understand the dose-dependent effect of GA on the biomarkers of four molecular pathways involved in liver toxicity. The serum levels of GA for each dose were calculated using Cmax value [36] and are shown in Table 1 below. The serum values of GA were assumed to be same as the mean liver values for GA.

The simulations were performed for a period of two days as all the model output parameters reached a steady state value within that period. GA was administered at the beginning of the simulations, starting at *t* = 0 s, and was maintained at same concentration levels for the duration of the simulations. 

The following computer simulations are performed:

1. Effect of GA on ROS levels
2. Effect of GA on glucuronide deconjugate levels
3. Effect of GA on C-PARP levels
4. Effect of GA on β-glucuronidase synthesis

## 3. Results

This study provides three results: (1) A curated set of literature related to liver toxicity, (2) the identification of molecular pathways involved in the liver toxicity, and (3) in silico efficacy analysis of effect of GA on four molecular pathways involved in liver toxicity.

### 3.1. Systematic Literature Review 

A systematic literature review resulted in the identification of an initial set of 119 articles (duplicates were removed) (see Figure 1 for the PRISMA study selection flow chart). Further analysis of the title and abstract yielded 93 relevant articles that were comprehensively reviewed by the authors. Of these 85 relevant articles, 45 informed about the 4 molecular pathways related to liver toxicity, 29 informed about the biochemical interactions between phytonutrients and the molecular pathways related to liver toxicity, and 19 informed about the pharmacokinetic and pharmacodynamics properties of GA.

### 3.2. Molecular Pathways Involved in Liver Toxicity

Four pathways including ROS production pathway, deconjugation/deglucuronidation pathway, hepatic apoptosis pathway, and β-glucuronidase synthesis were identified to be involved in liver toxicity. They are described in detail below.

#### 3.2.1. ROS Production Pathway Involved in Liver Toxicity 

**Figure 2:**
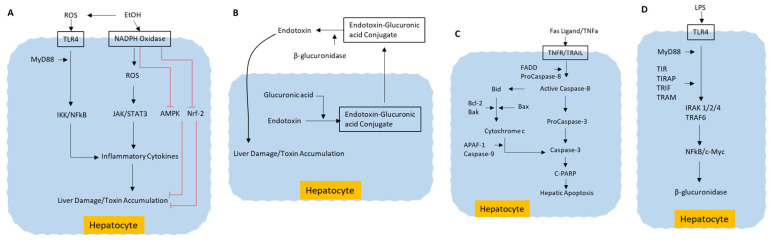
Molecular pathways of liver toxicity. (**A**) ROS production resulting from exogenous toxins, such as alcohol, inducing liver damage by apoptosis; (**B**) Liver damage caused by deconjugation of endotoxins; (**C**) Death receptor-mediated signaling pathway in liver cells inducing liver damage by apoptosis; (**D**) β-glucuronidase synthesis pathway induced by endotoxin LPS. EtOH—Ethanol; ROS—Reactive oxygen species; TLR4—Toll like receptor 4; NADPH—Nicotinamide adenine dinucleotide phosphate; MyD88—Myeloid differentiation primary response 88; IKK—IκB kinase; NFkB—Nuclear factor kappa-light-chain-enhancer of activated B cells; JAK—Janus kinase; STAT3—Signal transducer and activator of transcription 3; AMPK–5’—AMP-activated protein kinase; Nrf-2—Nuclear factor erythroid 2–related factor 2; TNFa—Tumor necrosis factor-alpha; TNFR—TNF receptor; TRAIIL—TNF-related apoptosis-inducing ligand; FADD—Fas-associated death domain; Bid—BH3 interacting domain; Bcl2—B-cell CLL/lymphoma 2; Bax—Bcl2-associated X protein; Bak—Bcl-2 homologous antagonist/killer; APAF-1—apoptosis-activating factor-1; C-PARP—cleaved Poly (ADP-ribose) polymerase; LPS—Lipoplysaccharide; MyD88—Myeloid differentiation primary response 88; TLR4—Toll-like receptor 4; TIRAP—TIR domain containing adaptor protein; TRIF—TIR domain-containing adaptor inducing IFN-β; TRAM—TRIF related adaptor molecule; IRAK 1/2/4—IL-1R-associated kinases 1/2/4; TRAF6—TNF-receptor-associated factor 6; NF-κB—nuclear factor kappa-light-chain-enhancer of activated B cells; c-Myc—Cellular myelocytomatosis.

Hepatotoxicity induced by exogenous toxins such as alcohol [18] and carbon tetrachloride (CCl4) is mediated through ROS production, which leads to apoptosis in liver cells [37,38,39]. In Kupffer cells, LPS interacts with Toll-like 4 (TLR-4) receptor and begins a signaling mechanism that facilitates liver damage by favoring the production of ROS production and pro-inflammatory cytokines. Reactive oxygen species (ROS) produced by cytochrome P450 2E1 (CYP2E1) and nicotinamide adenine dinucleotide phosphate (NADPH+) oxidase activity aggravate the cell response to endotoxins by highly increasing the transduction of signals mediated by TLR-4 through transcription factors, such as the nuclear factor kappa-B (NF-κB) and STAT3. Additionally, ROS induced by alcohol has been shown to inhibit hepatoprotective adenosine monophosphate activated protein kinase (AMPK) [17]. The inhibition of nuclear factor erythroid 2-related factor-2 (Nrf2) by ROS is implicated in the accumulation of liver toxins [18,40]. The various signaling pathways that are involved in exotoxin- and alcohol-induced ROS production and ROS-induced liver toxicity are schematically represented in Figure 2A.

#### 3.2.2. Deconjugation/Deglucuronidation Pathway in Liver Toxicity

Glucuronidation of several endotoxins is an important part of phase-II conjugation reaction and is catalyzed by the enzyme UDP glucuronate β-D-glucuronosyltransferase (UDPGT). The toxins from intestinal bile or liver will form glucuronide conjugates by affixing to glucuronic acid. These conjugates are excreted in the bile and urine or transported from liver to other tissues [41]. The opposite reaction to this is deglucuronidation, the deactivation and elimination of glucuronide conjugates. This reaction will mediate reabsorption of conjugate moieties in liver, instead of its excretion, which will increase liver toxicity. Deglucuronidation is catalyzed by β-glucuronidase enzyme [42]. Hydrolysis of the glucuronide moiety can be carried out by β-glucuronidase present in most tissues, particularly liver, kidney, spleen, intestinal epithelium, and endocrine and reproductive organs [43]. Thus, circulating inactive glucuronyl conjugates that are destined for excretion are now recognized as potential toxins at the target tissues. Thus, inhibition of β-glucuronidase activity in the liver can be a hepatoprotective mechanism, thus preventing liver damage due to toxicity. The schematics of this pathway are represented in Figure 2B.

#### 3.2.3. Hepatic Apoptosis Pathway in Liver Toxicity 

One of the key hepatotoxic mechanisms is hepatic apoptosis, which is mediated by TNF-α or Fas through mitochondrial activation pathways [44]. In hepatocytes, factors such as acute ethanol administration may initiate apoptosis by increasing the amount of Fas protein expression. Upon binding to its death receptor, Fas undergoes oligomerization [45]. This leads to the recruitment of cytoplasmic adapter protein Fas-associated death domain (FADD). FADD contains a death effector domain which mediates the recruitment of caspase 8 and caspase 10. Activated caspase 8 and caspase 10 cleave the pro-apoptotic BH3 interacting domain (Bid) protein. This truncated Bid is translocated to mitochondria inducing release of cytochrome c from mitochondria. The cytosolic cytochrome c binds to apoptosis-activating factor-1 (Apaf-1), facilitating recruitment of caspase 9 in a protein complex. This apoptosome activates effector caspases such as caspase 3 and 7, which in turn bring about apoptosis-mediated liver damage [45,46] via cleaved poly (ADP-ribose) polymerase (cPARP). A brief schematic of this pathway is represented in Figure 2C.

#### 3.2.4. β-Glucuronidase Synthesis Pathway in Liver Toxicity 

Higher activity of β-glucuronidase promotes deconjugation of endotoxins and the formation of calcium bilirubinate in liver, which in turn increases hepatotoxicity-mediated liver damage [13,47]. Lipopolysaccharide (LPS), a major endotoxin from gram-negative bacteria induces, increased expression of endogenous β-glucuronidase in hepatocytes and intrahepatic biliary epithelial cells [13]. The LPS signaling proceeds via its binding to TLR4 and subsequent dimerization of TLR4. Homodimerized TLR4 induces the recruitment of adaptor proteins containing Toll/interleukin-1 receptor-like (TIR) domains. The engagement of adaptor molecules, such as myeloid differentiation primary response protein 88 (MyD88), TIR domain containing adaptor protein (TIRAP), TIR domain-containing adaptor inducing IFN-β (TRIF), and TRIF related adaptor molecule (TRAM), stimulates the recruitment of IL-1R-associated kinases (IRAKs). The formation of a complex of IRAK4, IRAK1, IRAK2, and TNF-receptor-associated factor 6 (TRAF6) leads to dissociation and activation of TRAF6. Activation of TRAF6 leads to activation of c-Myc gene via NFkB, which in turn induces the expression of β-glucuronidase. This molecular pathway is illustrated in Figure 2D.

### 3.3. Simulation Results 

The effect of GA was tested on four molecular pathway models, and the results are discussed in detail below.

#### 3.3.1. Effect of Glucaric Acid on ROS Production 

**Figure 3:**
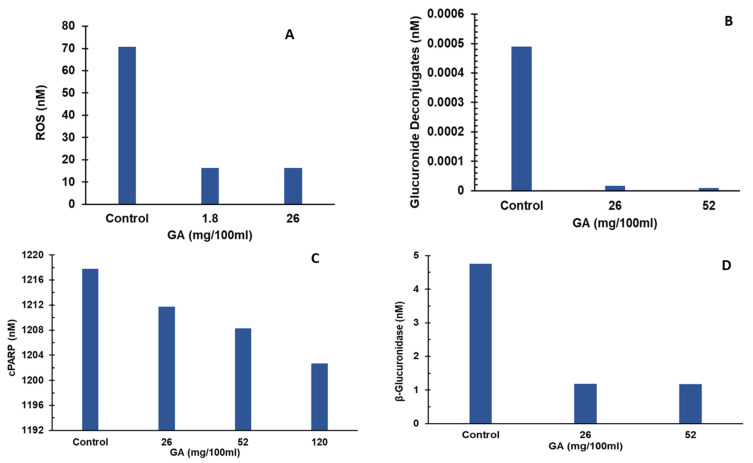
Effect of GA on liver toxicity pathways. (**A**) Effect of GA on ROS levels. GA supplementation led to reduction in reactive oxygen species concentrations compared to control. (**B**) Effect of GA on glucuronide deconjugates levels. GA supplementation led to reduction in glucuronide deconjugate concentrations compared to control. (**C**) Effect of GA on cPARP levels. GA supplementation led to a reduction in cPARP concentrations compared to control. (**D**) Effect of GA on β-glucuronidase levels. GA supplementation led to reduction in β-glucuronidase concentrations compared to control. GA—Glucaric acid; ROS—Reactive oxygen species; cPARP—cleaved poly (ADP-ribose) polymerase.

The effect of GA was simulated on ROS production pathway by estimating the ROS levels in hepatocytes over a period of two days. ROS levels were induced by alcohol toxicity in the simulations. GA supplementation levels used in the simulations were 0, 1.8, and 26 mg. Under control conditions, the system was assumed to be in a state of alcohol induced liver toxicity condition with no GA supplementation, and the ROS levels were estimated to be 70 nM. Increasing the GA supplementation to 1.8 mg led to a significant decrease in ROS levels of 16 nM at the end of simulation period, as shown in Figure 3A. These results substantiate the role of GA as hepatoprotective via lowering ROS. ROS production is implicated in promoting expression of proinflammatory cytokines that cause liver damage and liver toxicity [17,18]. An increase in GA supplementation to 26 mg, 52 mg, and 120 mg did not lower ROS levels any further.

#### 3.3.2. Effect of Glucaric Acid on Deconjugation/Deglucuronidation 

The effect of GA was simulated on the deconjugation pathway by estimating the glucuronide deconjugate levels in the bile over a period of two days. Simulations were conducted for GA supplementation levels of 0, 26 mg, and 52 mg. Under control conditions, the system was assumed to be in an infectious state with elevated endotoxin levels with no glucaric acid supplementation, and the glucuronide deconjugate levels were estimated to be 4.9 × 10^−3^ nM. Increasing the GA supplementation to 26 mg led to a significant decrease in glucuronide deconjugate levels to 1.7 × 10^−5^ nM at the end of the simulation period, as shown in Figure 3B. These results indicate that GA plays a hepatoprotective role by lowering glucuronide deconjugate levels, which are implicated in promoting liver toxicity and subsequent liver damage [19,48]. This can be attributed to GA’s inhibition of β-glucuronidase which catalyzes the deconjugation of endotoxin-glucuronic acid complexes. An increase in GA supplementation to 52 mg further lowered glucuronide deconjugate levels to 8.9 × 10^−6^ nM. GA supplementation of 1.8 mg did not lower glucuronide deconjugate levels as compared to the control value. A further increase in GA supplementation to 52 mg did not lower glucuronide deconjugate levels any further.

#### 3.3.3. Effect of GA on Hepatic Apoptosis 

GA was shown to have a negative regulatory effect on the activation of pro-apoptotic Caspase-8 by increasing the Bcl-2 to Bax ratio [12]. To analyze the effect of GA on hepatic apoptosis, we estimated cPARP (a marker of apoptosis) levels at varying GA levels over a period of two days. Under control conditions, the system was assumed to be in an infectious state with elevated pro-inflammatory cytokines simulating a pro-apoptotic environment with estimated cPARP levels of 1217 nM. As shown in Figure 3C, GA dose-dependently lowered cPARP levels to 1211, 1208, and 1202 nM for GA supplementation of 26, 52, and 120 mg, respectively. These results show a moderate but consistent effect on lowering of cPARP, indicating a moderate role for GA in reducing hepatic apoptosis. The GA dose level of 1.8 mg did not reduce the cPARP level compared to the control value.

#### 3.3.4. Effect of GA on β-Glucuronidase Synthesis 

The effect of GA was simulated on the β-glucuronidase synthesis pathway by estimating the β-glucuronidase levels in the hepatocyte over a period of two days. Simulations were conducted for GA supplementation levels of 0, 26 mg, and 52 mg. Under control conditions, the system was assumed to be in an infectious state with elevated endotoxin levels with no GA supplementation, and the β-glucuronidase levels were estimated to be 4.75 nM. Increasing the GA supplementation to 26 mg led to a significant decrease in β-glucuronidase levels to 1.19 nM at the end of simulation period, as shown in Figure 3D. These results indicate that GA plays a hepatoprotective role by lowering β-glucuronidase levels, which is implicated in promoting the accumulation of toxins and subsequent liver damage [13,47,49]. A further increase in GA supplementation to 52 mg did not lower ROS levels any further.

## 4. Discussion

**Figure 4:**
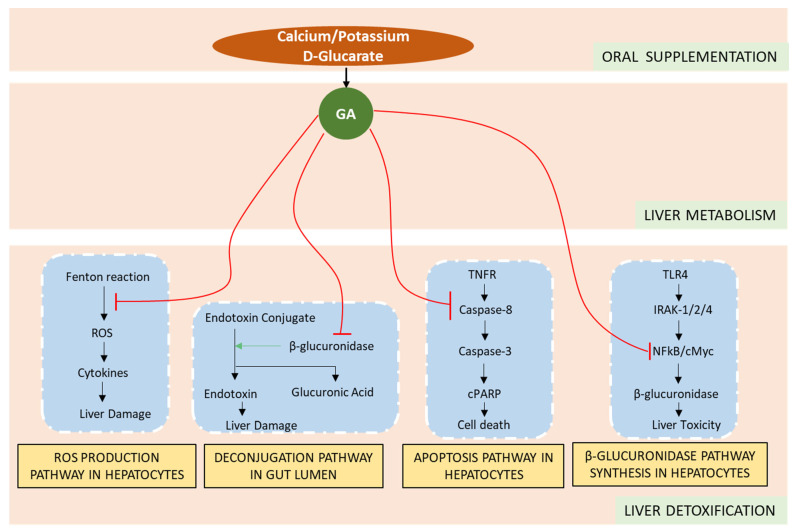
Molecular interactions of glucarate salt active metabolites, GA, on the four mechanisms of liver detoxification.

A systems biology approach was used to uncover mechanisms of how metabolites of glucaric salts may modulate molecular pathways of liver detoxification, as illustrated in Figure 4. 

These molecular mechanisms using CytoSolve were integrated to develop a computational framework for performing in silico experiments to quantify the effects of GA on specific biomarkers of liver toxicity across four major mechanisms, which, to our knowledge, is the first study of its kind to have applied such a computational system biology approach. Our results show that the metabolite of glucarate salts, GA, mitigate liver toxicity by: (1) reducing ROS production in hepatocytes, (2) lowering hepatic apoptosis, (3) reducing β-glucuronidase in hepatocytes, and (4) reducing glucuronide deconjugate levels in the bile. 

Previous studies have shown that GA affects Fenton reaction, a critical step in formation of ROS such as H_2_O_2_ and hydroxyl radical, by chelating Fe^3+^ [50]. Our results show that GA supplementation significantly reduced the ROS production via lowering the ROS levels in hepatocytes. These results are consistent with those reported by Subramanian and Madras [50], and furthermore explain the mechanism behind the hepatoprotective effects of GA. 

GA has been shown to competitively inhibit β-glucuronidase, which, in the gut lumen, promotes toxicity and generates carcinogenic substances [51]. In this study, we have shown that supplementation of GA significantly reduces the deconjugation of toxic substances and consequently lowers the toxicity in liver in a dose-dependent manner. DSL, a GA metabolite, has been shown to effectively inhibit the transcription factor NF-kB, which enables the expression of hepatotoxic β-glucuronidase [13,47]. Results from this study indicate a significant reduction in β-glucuronidase with increased supplementation of GA. 

The study herein models the effect”of G’ at the cellular level. Given the main purpose of this study is to derive a molecular mechanistic understanding of GA’s presence on liver detoxification, the in silico predications herein provide guidance for future in vitro and in vivo studies.

## 5. Conclusions and Future Work

### 5.1. Conclusions 

In conclusion, to our knowledge, this is the first study of its kind that identifies the critical mechanisms of action behind the mitigation of liver toxicity by GA observed in experimental studies. Results from this study show that GA mitigates liver toxicity by downregulating ROS production, suppressing the deconjugate accumulation, inhibiting hepatic apoptosis, and reducing β-glucuronidase synthesis. Additionally, this study provides a framework for future research to further our understanding of how other nutrients can individually, or in combination with GA, support liver detoxification and improve muscle health, as well as other liver pathologies such as hepatitis, cirrhosis of liver, and non-alcoholic steatohepatitis. 

### 5.2. Future Work 

While the current model provides mechanistic explanation for experimental observations [12,13,17,18,47,49], future in vitro or in vivo experimental studies can serve to further strengthen the conclusions from this study. Additionally, the liver toxicity model is currently based on four pathways: ROS production pathway, deconjugation/deglucuronidation pathway, hepatic apoptosis pathway, and β-glucuronidase synthesis. The modular computational framework afforded by the study allows for ongoing expansion and integration of other relevant pathways. For example, the molecular pathway of liver toxicity induced by mitochondrial dysfunction [52] could be integrated to expand the liver toxicity model and enhance its robustness. The interactions of derivatives of GA such as DSL can also be explored in future work.

## Figures and Tables

**Table 1:** Glucaric acid doses and their respective serum concentrations.

Glucaric Acid Dose (mg/100 mL)	Serum Concentration (µM)
1.8	2.7
26	39
52	78
120	180

## Data Availability

Not applicable.

## Supplementary Materials

The following supporting information can be downloaded at: https://www.mdpi.com/article/10.3390/nu15030733/s1, Supplementary File S1: CytoSolve® Operating Guide Protocol Summary; Supplementary File S2: CytoSolve Computational Modeling of Liver Detoxification Pathways; Supplementary File S3: Medical Subject Heading (MeSH) Keywords. Refs. [49,53,54,55,56,57,58,59,60,61,62,63,64,65,66,67,68,69,70,71,72,73,74,75,76,77,78,79,80,81,82,83,84,85,86,87,88,89,90,91,92,93,94,95,96,97] are cited in supplementary materials.
