# Supplementary material for: Mechanistic Understanding of D-Glucaric Acid to Support Liver Detoxification Essential to Muscle Health Using a Computational Systems Biology Approach
Source: Nutrients. 2023 Feb 1;15(3):733. doi: 10.3390/nu15030733 (PMC9921405; doi:10.3390/nu15030733)
Supplement: Supplementary file 1 [file nutrients-15-00733-s001.zip › nutrients-2075473-supplementary.pdf]

Mechanistic Understanding of D-Glucaric Acid to Support Liver Detoxification Essential to Muscle Health Using a Computational Systems Biology Approach

V.A. Shiva Ayyadurai, Prabhakar Deonikar, and Christine Fields

Supplementary Information

Supplementary File S1
CytoSolve® Operating Guide Protocol Summary

1.1. CytoSolve® System

CytoSolve is a well-established computational systems biology framework of technology and processes that provides the capability to derive molecular mechanisms of action; to create quantitative and predictive models of those mechanisms; and, to employ the resultant models to simulate complex biomolecular phenomena [1–6]. In neurovascular studies, the CytoSolve framework elicited and derived a multi-layered engineering molecular systems architecture integrating the anatomy of the neurovascular unit, molecular mechanisms, and disease to demonstrate the commonality of multiple neurovascular diseases as communication dysfunctions in common molecular signaling sub-systems and compounds [4].

In oncology, CytoSolve's capability has been employed for the *in silico* modeling of pancreatic cancer to identify and optimize a multi-combination therapeutic that was subsequently allowed for clinical trials by the United States Food and Drug Administration [7], has been used to identify the molecular systems architecture of interactome in acute myeloid leukemia (AML) microenvironment [8], and has been independently recognized by leading cancer researchers as a

platform for developing multi-combination therapies [3]. In cardiovascular research, CytoSolve has been used to accurately model the release of nitric oxide (NO) production in endothelial cells subjected to shear stress [5].

In the area of plant biology, CytoSolve enabled the quantitative molecular systems understanding of C1 metabolism - a critical system of molecular pathways inherent to all plants, fungi and bacteria - to understand the systemic effects oxidative stress and genetic modification on C1 metabolism in soy [9–12]. Recently, CytoSolve was used to discover and model the mechanisms of immunomodulatory effect of bioactive compound in green tea on organ transplant tolerance [13], and elucidate effect of bioactive compounds from fruit, berry, vegetable (FBV) juice power on low grade chronic inflammation [14].

Details of CytoSolve® Operating Guide Protocol can be found in previous publications [14]

1.2. CytoSolve® System Capabilities

The method used in this study provides a scalable computational framework for modeling large-scale biological systems by dynamic integration of an ensemble of multiple molecular pathway models [1]. This method enables the development of large-scale models of complex biological systems that span multiple temporal and spatial scales as well as across diverse domains. Rather than attempting to monolithically model systems of biochemical reactions, a distributed engineering systems approach – a relatively novel concept in systems biology– is employed that breaks a large scale biological system into an ensemble of smaller molecular pathway models that

are computationally coupled. This approach makes the modeling of large-scale biological systems both tractable and scalable.

1.3. Key Elements of CytoSolve® System Protocol

There are six (6) steps that comprise the protocol to use the CytoSolve® system. Figure S1 illustrates those steps of the protocol.

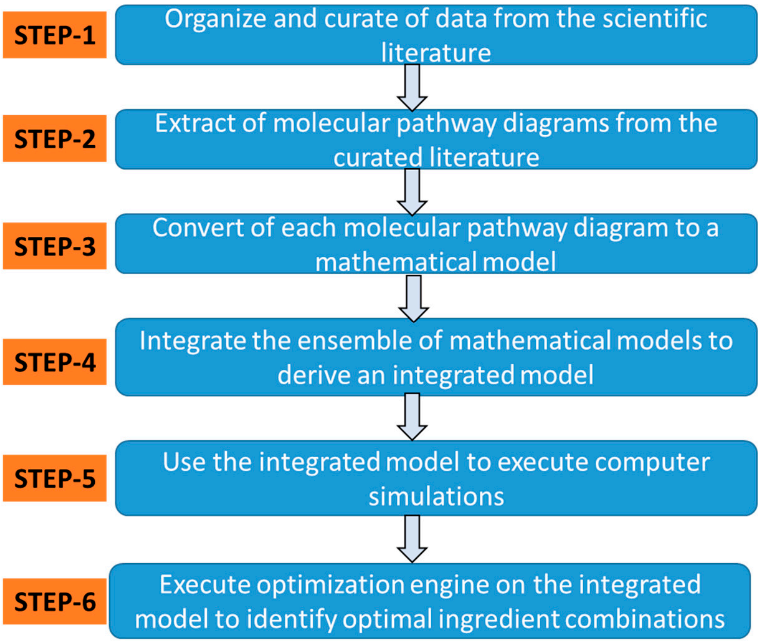

Figure S1: CytoSolve® Protocol Overview. The six steps involved in the CytoSolve® protocol. Steps 1 and 2 relate to performing systematic literature review to identify molecular pathways and the biochemical parameters required for computational modeling. Steps 3 to 5 relate to construction of individual models, integration of individual models, and executing simulations using the integrated modes. Step 6 provides the option to employ CytoSolve® optimization engine to discover the optimal combination of inputs (ingredients/compounds) for a specific range of values of outputs (biomarkers associated with particular biomolecular functions).

The six (6) steps are listed below:

1) Organize and curate data from the scientific literature

2) Extract molecular pathway diagrams from the curated literature

3) Convert each molecular pathway diagram to a mathematical model

4) Integrate individual mathematical models to derive an integrated model

5) Use the integrated model to execute computer simulations to analyze the effect of ingredients on interest, individually as well as in combination

6) Execute optimization engine on the integrated model to identify optimal ingredient combination

1.4. CytoSolve® System Limitations

Although the framework developed in this study provides a detailed mechanistic understanding of liver toxicity that match well with published clinical data, some of the model components' parameters were derived from experiments using different cell types, as well as different experimental conditions such as variations in culture conditions that adds to the uncertainty of the model predictions [15]. Such issues of parameter estimation, however, are not unique to this study. They are common to a number of cellular mathematical models [15] and do warrant further experimental investigation and validation.

1. Ayyadurai, V.A.S.; Dewey, C.F. CytoSolve: A Scalable Computational Method for Dynamic Integration of Multiple Molecular Pathway Models. *Cell. Mol. Bioeng.* **2011**, *4*, 28–45.
2. Nordsletten, D.A.; Yankama, B.; Umeton, R.; Ayyadurai, V.V.S.; Dewey, C.F. Multiscale Mathematical Modeling to Support Drug Development. *IEEE Trans. Biomed. Eng.* **2011**, *58*, 3508–3512.
3. Al-Lazikani, B.; Banerji, U.; Workman, P. Combinatorial drug therapy for cancer in the post-genomic era. *Nat. Biotechnol.* **2012**, *30*, 679–92.
4. Sweeney, M.D.; Ayyadurai, S.; Zlokovic, B. V. Pericytes of the neurovascular unit: key functions and signaling pathways. *Nat. Neurosci.* **2016**, *19*, 771–783.
5. Koo, A.; Nordsletten, D.; Umeton, R.; Yankama, B.; Ayyadurai, S.; García-Cardeña, G.; Dewey, C.F. In Silico Modeling of Shear-Stress-Induced Nitric Oxide Production in Endothelial Cells through Systems Biology. *Biophys. J.* **2013**, *104*, 2295–2306.

6. Ayyadurai, V.A.S.; Deonikar, P. In Silico Modeling and Quantification of Synergistic Effects of Multi-Combination Compounds: Case Study of the Attenuation of Joint Pain Using a Combination of Phytonutrients. *Appl. Sci. 2022, Vol. 12, Page 10013* **2022**, *12*, 10013.
7. Food and Drug Administration Center for Drug Evaluation and Research Request for Determination of Exempt Status of Investigational New Drug Application (IND) for Cyto-001 as Treatment for Patients with Pancreatic Cancer (PIND: 118833) 2013.
8. Ayyadurai, V.A.S.; Deonikar, P.; McLure, K.G.; Sakamoto, K.M. Molecular Systems Architecture of Interactome in the Acute Myeloid Leukemia Microenvironment. *Cancers 2022, Vol. 14, Page 756* **2022**, *14*, 756.
9. Ayyadurai, V.A.S.; Deonikar, P. Do GMOs Accumulate Formaldehyde and Disrupt Molecular Systems Equilibria? Systems Biology May Provide Answers. *Agric. Sci.* **2015**, *06*, 630–662.
10. Kothandaram, S.; Deonikar, P.; Mohan, M.; Venugopal, V.; Ayyadurai, V.A.S. <i>In Silico</i> Modeling of C1 Metabolism. *Am. J. Plant Sci.* **2015**, *06*, 1444–1465.
11. Mohan, M.; Kothandaram, S.; Venugopal, V.; Deonikar, P.; Ayyadurai, V.A.S. Integrative Modeling of Oxidative Stress and C1 Metabolism Reveals Upregulation of Formaldehyde and Downregulation of Glutathione. *Am. J. Plant Sci.* **2015**, *06*, 1527–1542.
12. Ayyadurai, V.A.S.; Hansen, M.; Fagan, J.; Deonikar, P. In-Silico Analysis & In-Vivo Results Concur on Glutathione Depletion in Glyphosate Resistant GMO Soy, Advancing a Systems Biology Framework for Safety Assessment of GMOs. *Am. J. Plant Sci.* **2016**, *07*, 1571–1589.
13. Ayyadurai, V.A.S.; Deonikar, P. Bioactive compounds in green tea may improve transplant tolerance: A computational systems biology analysis. *Clin. Nutr. ESPEN* **2021**, *46*.
14. Ayyadurai, V.A.S.; Deonikar, P.; Bannuru, R.R. Attenuation of low-grade chronic inflammation by phytonutrients: A computational systems biology analysis. *Clin. Nutr. ESPEN* **2022**.
15. Thanh, V.H.; Zunino, R.; Priami, C. Efficient finite-difference method for computing sensitivities of biochemical reactions. *Proc. R. Soc. A Math. Phys. Eng. Sci.* **2018**, *474*.

Supplementary File S2

CytoSolve Computational Modeling of Liver Detoxification Pathways

Details of mathematical models used to assess the effect of glutamic acid (GA) on liver toxicity are given below. Four mathematical models were constructed to represent four different signaling transduction pathways involved in liver toxicity. For each of the models, section below lists the initial conditions for the parameters, biochemical reactions, the corresponding rate equations, and the rate constants.

Section 2.1 contains the information used to model the oxidative stress pathway involved in the liver toxicity in hepatic cells, which leads to formation of oxidative stress biomarker reactive oxygen species (ROS).

Section 2.2 contains the information used to model the deconjugation/deglucuronidation pathway involved in the liver toxicity in hepatic cells, which leads to formation of the biomarker glucuronide deconjugate.

Section 2.3 contains the information used to model the hepatic apoptosis pathway involved in the liver toxicity in hepatic cells, which leads to formation of apoptotic biomarker cPARP.

Section 2.4 contains the information used to model the β-glucuronidase synthesis pathway involved in the liver toxicity in hepatic cells, which leads to formation of the biomarker β-glucuronidase.

Additional literature was identified to obtain biochemical reactions, kinetic parameters and initial conditions to simulate the in silico models and is available in the bibliography of the Supplementary Information 2.

2.1. In Silico Modeling of Oxidative Stress in Hepatocytes

Table S1: Initial Concentrations

Species	Value (nM)	Ref
LH	351000.0	(Babbs & Steiner, 1990)
O2	10000.0	(Atunes et al., 1996)
H2O	5.5E7	(Shi et al., 2013)
Fe2+	100.0	(Atunes, Salvador, Marinho, Alves, & Pinto, 1996)
Fe3+	6800.0	(Ambrosio et al., 1987)
SOD	700.0	(Kavida, 2011)
H2O2	0.38	(Atunes et al., 1996)
Catalase	41.03	(Aydemir & Kuru, 2003)
GSH	1000.0	(Shi et al., 2013)
GPr	1.0	(Shi et al., 2013)

Glossary:
LH – Lipid peroxide
O_2 – Oxygen
H_2O – Water
Fe^{2+} – Ferrous ion
Fe^{3+} – Ferric ion
SOD – Superoxide dismutase
H_2O_2 – Hydrogen peroxide
GSH – Glutathione
GPr – Glutathione peroxidase

Table S2: Biochemical Reactions and their Rate Equations

Biochemical Reaction	Rate Equation
[L·] + [O2] → [LOO·]	kLPO × [L·] x [O2]
[LH] + [LOO·] → [LOOH] + [L·]	[LH] × [LOO] · × kLR1
[LOOH] + [Fe2+] ↔ [LO·] + [Fe3+] + [OH-]	[LOOH] × [Fe2+] × kLRFe1

[LOOH] + [Fe3+] ↔[LOO·] + [Fe2+] + [H+]	[LOOH] × [Fe3+] × kLRFe2
[H2O2] + [Catalase] →[H2O] + [O2]	[H2O2] × [Catalase] × kcat
[LH] + [LO·] ↔ [LOH] + [L·]	[LH] × [LO·] × kLR2
[GPo] + [GSH] ↔[GSGP] + [GSSG] + [H20]	[GPo] × [GSH] × kGSGP
[GSH] + [GSGP] ↔[Gpr] + [GSSG] + [H20]	[GSH] × [GSGP] × kGPr
[H2O2] + [GPr] →[H2O] + [GPo]	[H2O2] × [H+] * [H+] × [Gpr] × kGPo
[H+] + [O2-·] + [SOD] →[H2O2]	[H+] × [O2-]· × [SOD] × [kSOD] × 0.25
[H2O] ↔ [H+]+ [OH]	[H2O] × kdH2O - [H+] × [OH-] × [KH2O]
[LH] + [OH·] ↔[L·] + [H2O]	[LH] × [OH] × kinitLR
[H2O2] + [Fe2+] ↔[Fe3+] + [OH·] + [OH-]	[Fe2+] × [H2O2] × kFe1
[OH·] + [Fe2+] ↔[Fe3+] + [OH-]	[OH] × [Fe2+] × kFe6
[Fe3+] + [H2O2]↔[Fe2+] + [HO2·] + [H+]	kFe5 × [Fe3+] × [H2O2]
[HO2·] + [Fe3+] ↔[Fe2+] + [O2] + [H+]	[HO2] × [Fe3+] × kFe3
[O2-*] + [Fe3+] ↔[Fe2+] + [O2]	[O2-·] × [Fe3+] × kFe3
[H2O2] + [OH*] ↔ [HO2·] + [H2O]	[H2O2] × [OH·] × kFe4
[OH·] + [HO2·] ↔[H2O] + [O2]	[OH·] × [HO2·] ×kFe7
[HO2·*] + [H2O2]↔[OH·] + [H2O] + [O2]	[HO2] · × [H2O2] × kFe9
[HO2·] + [HO2·] ↔ [H2O2]	[HO2] · × [HO2] × kFe8
Φ ↔ [O2-·]	kstress_HX × scale1 × [Factor] / (1 + kmstress_HX) - [O2-·] × kdegROS
[Glucaric acid] + [Fe3+] → [Glucaric acid_Fe3+]	[Glucaric acid] × [Fe3+] × kSA_fe3
[Glucaric acid_Fe3+] → [Glucaric acid] + [Fe3+]	[Glucaric acid_Fe3+] × kdSAFeIII

Table S3: Chemical Kinetic Parameters

Parameter	Value	Units	Reference
kinitLR	0.5	$nM^{-1}\ s^{-1}$	(Atunes et al., 1996)
kLPO	0.3	$nM^{-1}\ s^{-1}$	(Atunes et al., 1996)
kLR1	1.4×10^{-8}	$nM^{-1}\ s^{-1}$	(Atunes et al., 1996)
kFe1	7.6×10^{-8}	$nM^{-1}\ s^{-1}$	(Henle et al., 1996)
kFe6	0.35	$nM^{-1}\ s^{-1}$	(Henle et al., 1996)

kFe3	3.1×10^{-4}	$nM^{-1}\ s^{-1}$	(Henle et al., 1996)
kLR2	0.0066	$nM^{-1}\ s^{-1}$	(Atunes et al., 1996)
kLRFe1	0.015	$nM^{-1}\ s^{-1}$	(Xue et al. 2012)
kLRFe2	1.0×10^{-6}	$nM^{-1}\ s^{-1}$	(Xue et al., 2012)
kGPo	0.021	$nM^{-1}\ s^{-1}$	(Buettner, Ng, Wang, Rodgers, & Schafer, 2006)
kGSGP	4.0×10^{-5}	$nM^{-1}\ s^{-1}$	(Buettner et al., 2006)
kGPr	0.01	$nM^{-1}\ s^{-1}$	(Buettner et al., 2006)
kSOD	1.6	$nM^{-1}\ s^{-1}$	(Edwards, Cao, & Pallone, 2011)
kcat	0.034	$nM^{-1}\ s^{-1}$	(Edwards, Cao, and Pallone 2011)
KH2O	140.0	$nM^{-1}\ S^{-1}$	(H.Stillinger, 1978)
kdH2O	2.5×10^{-5}	S^{-1}	(H.Stillinger, 1978)
kFe2	0.0012	$nM^{-1}\ s^{-1}$	(Henle et al., 1996)
kFe4	0.027	$nM^{-1}\ s^{-1}$	(Henle et al., 1996)
kFe5	2.7×10^{-10}	$nM^{-1}\ s^{-1}$	(Henle et al., 1996)
kFe7	7.0	$nM^{-1}\ s^{-1}$	(Henle et al., 1996)
kFe8	0.017	$nM^{-1}\ s^{-1}$	(Henle et al., 1996)
kFe9	5.0×10^{-10}	$nM^{-1}\ s^{-1}$	(Henle et al., 1996)
kFe10	5.5	$nM^{-1}\ s^{-1}$	(Henle et al., 1996)
kstress_HX	166666.66	$nM\ S^{-1}$	(Taylor, Styles, Matthews, & Gadian, 1986)
kmstress_HX	30000	nM	(Taylor et al. 1986)
kdegROS	0.085	S^{-1}	Estimated (Macfarlane & Miller, 1992)
kSA_fe3	0.154	S^{-1}	(Subramanian & Madras, 2016)
kdSAFeIII	1.15×10^{-4}	S^{-1}	(Subramanian and Madras 2016)

2.2. In Silico Modeling of Deconjugation/Deglucuronidation Pathway

Table S4: Initial Concentrations

Species	Value (nM)	Ref
Glucuronic acid	530	(Aw & Jones, 1982)
β-Glucuronidase	169.57	(Feng et al., 1997)
Endotoxin_E	0.106	(Harte et al., 2010)

Table S5: Biochemical Reactions and their Rate Equations

Biochemical Reaction	Rate Equation
[Glucuronic conjugate]→[Glucuronide deconjugate] + [Glucuronic conjugate]	[Glucuronic conjugate] × [β-Glucuronidase] × kcat_ben_bgluc / (kmben_bglucd × (1 + [GA] / kiGA_BGA) + [Glucuronic conjugate])
[Endotoxin_E] → [Endotoxin_C]	[Endotoxin_E] × ktransEndotoxin / (kmtrans + [Endotoxin_E])
[Endotoxin_C] + [Glucuronic acid]→[Glucuronic conjugate]	[Endotoxin_C] × [Glucuronic acid] × Vben_pyr_glu / (kmben_pyr_glu + [Endotoxin_C] × [Glucuronic acid])
[Glucuronic conjugate] → Φ	[Glucuronic conjugate] × kdeg_gluc
[Glucuronide deconjugate] → Φ	[Glucuronide deconjugate] × kdeg_degluconju

Table S6 Chemical Kinetic Parameters

Parameter	Value	Units	Reference
ktransEndotoxin	0.043	$nM^{-1}S^{-1}$	(Nolan et al. 1977)
kmtrans	28333.33	nM	(Nolan et al. 1977
kiGA_BGA	1600	nM	(Dwivedi et al., 1990)
Vben_bglucd	25.33	$nM^{-1}S^{-1}$	(Tomasic & Keglevic, 1973)
kmben_bglucd	85000	nM	(Tomasic & Keglevic, 1973)

Vben_pyr_glu	0.47833	$nM^{-1}S^{-1}$	(Nichols et al. 2017)
kmben_pyr_glu	30	nM	(Nichols et al., 2017)
kdeg_gluc	1.003×10^{-4}	S^{-1}	(Boase & Miners, 2002)
kdeg_degluconju	1.45×10^{-5}	S^{-1}	Estimated
kcat_ben_bgluc	0.1494	S^{-1}	Calculated from (Tomasic & Keglevic, 1973)

2.3. In Silico Modeling of Hepatic Apoptosis Pathway

Table S7: Initial Concentrations

Species	Value (nM)	Reference
FLIP	0.17	(Albeck, Burke, Spencer, Lauffenburger, & Sorger, 2008)
C8	34	(Albeck et al. 2008)
BAR	1.7	(Albeck et al. 2008)
C3	17	(Albeck et al. 2008)
C6	17	(Albeck et al. 2008)
XIAP	170	(Albeck et al. 2008)
PARP	1700	(Albeck et al. 2008)
Bid	68	(Albeck et al. 2008)
BCL-2c	34	(Albeck et al. 2008)
BAX	170	(Albeck et al. 2008)
BCl-2	480	(Albeck et al. 2008)
M	12000	(Albeck et al. 2008)
CyCm	12000	(Albeck et al. 2008)
SMAC m	2400	(Albeck et al. 2008)

APAF	170	(Albeck et al. 2008)
C9	170	(Albeck et al. 2008)
TNFR	0.1534	(Harte et al., 2010)
TRADD	91.7	(Schliemann et al., 2011)
TNF-alpha (Normal)	0.000289	(J. Li et al., 2014)
TNF-alpha (Liver Disease)	0.00126	(J. Li et al. 2014)
RIP1	63.3	(Schliemann et al. 2011)
TRAF2	103	(Schliemann et al. 2011)
FADD	96.7	(Schliemann et al. 2011)
FLIP	0.17	(Albeck, Burke, Spencer, Lauffenburger, & Sorger, 2008)
C8	34	(Albeck et al. 2008)
BAR	1.7	(Albeck et al. 2008)
C3	17	(Albeck et al. 2008)
C6	17	(Albeck et al. 2008)
XIAP	170	(Albeck et al. 2008)
PARP	1700	(Albeck et al. 2008)
Bid	68	(Albeck et al. 2008)
BCL-2c	34	(Albeck et al. 2008)
BAX	170	(Albeck et al. 2008)
BCl-2	480	(Albeck et al. 2008)
M	12000	(Albeck et al. 2008)
CyCm	12000	(Albeck et al. 2008)
SMAC m	2400	(Albeck et al. 2008)

APAF	170	(Albeck et al. 2008)
C9	170	(Albeck et al. 2008)
TNFR	0.1534	(Harte et al., 2010)
TRADD	91.7	(Schliemann et al., 2011)
TNF-alpha (Normal)	0.000289	(J. Li et al., 2014)
TNF-alpha (Liver Disease)	0.00126	(J. Li et al. 2014)
RIP1	63.3	(Schliemann et al. 2011)
TRAF2	103	(Schliemann et al. 2011)
FADD	96.7	(Schliemann et al. 2011)

Glossary:
FLIP - Fluorescence Loss in Photobleaching
C8 - Complement component 8
BAR - Bin/Amphiphysin/Rv
C3 - Complement component 3
C6 - Complement component 6
XIAP - X-linked inhibitor of apoptosis protein
PARP - Poly (ADP-ribose) polymerase
Bid - BH3-interacting domain death agonist
BCL-2c - B-cell lymphoma 2 cytochrome
BAX - BCL2 Associated X, Apoptosis Regulator
BCl-2 - B-cell lymphoma 2
CyCm - cyclin M
SMAC m - Second mitochondria-derived activator of caspase
APAF - Apoptotic protease activating factor 1
C9 - Complement component 9
TNFR - Tumor necrosis factor receptor
TRADD - TNFR type 1-associated death domain protein
TNF-α - Tumor necrosis factor-α
RIP1 - Receptor interacting protein-1
TRAF2 - TNF receptor-associated factor 2
FADD - Fas-associated protein with death domain

Table S8: Biochemical Reactions and their Rate Equations

Description	Rate Equation
[BAX2] + [BCl-2] ↔ [BAX2:BCL-2]	1 / v × [BAX2] × [BCl-2] × k16 - [BAX2:BCL-2] × k_16
[BAX4] + [BCl-2] ↔ [BAX4:BCl-2]	1 / v × [BAX4] * [BCl-2] × k18 – [BAX4:BCl-2] × k_18
[BAX4] ↔ [BAX4:M]	1 / v × [BAX4] × M × k19 - [BAX4:M] × k_19
[M·] + [CyCm] ↔ [M·:CyCm]	1 / v ×[M·] × [CyCm] × k20 - k_20 × [M·:CyCm]
[M·] + [SMAC m] ↔ [M·:SMACm]	1 / v × [M·] × [SMAC m] × k21 - k_21 × [M·:SMACm]
[BAX·m] + [BCl-2] ↔ [BAX·m:BCl-2]	1 / v × k14 × [BAX·m] × [BCl-2] - [BAX·m:BCl-2] × k_14
[BAX·m] ↔ [BAX2]	1 / v × k15 × [BAX·m] × [BAX·m] / (1 + DSL / kind_DSL_BCL2) - k_15 × [BAX2]
[BAX2] ↔ [BAX4]	1 / v × k17 × [BAX2] × [BAX2] - k_17 × [BAX4]
[Apop:C3] → [C3·] + [Apop]	[Apop:C3] × K25
[BAX·] ↔ [BAX·m]	[BAX·] × k13 - [BAX·m] × k_13
[C8·] + [BAR] ↔ [C8·:BAR]	[C8·] × [BAR] × k4 - k_4 × [C8·:BAR]
[C8·] + [C3] ↔ [C8·:C3]	[C8·] × [C3] × k5 - k_5 × [C8·:C3]
[C8·:C3] → [C3·] + [C8·]	[C8·:C3] × K5
[C8·:Bid] → [C8·] + [tBid]	K10 × [C8·:Bid]
[tBid:BAX] → [tBid] + [BAX·]	K12 × [tBid:BAX]
[BAX4:M] → [M·]	K19 × [BAX4:M]
[M·:CyCm] → [M·] + [CyCr]	K20 × [M·:CyCm]
[M·:SMACm] → [M·] + [SMACr]	K21 × [M·:SMACm]
[APAF:CyC] → [APAF·] + [CyC]	K23 × [APAF:CyC]

[C3·:C6] → [C6·] + [C3·]	K6 ×[C3·:C6]
[C6·:C8] → [C6·] + [C8·]	K7 × [C6·:C8]
[PARP:C3·] → [C3·] + [cPARP]	K9 × [PARP:C3·]
[R·C8]→ [TNFRC3] + [C8·]	[R·C8] × K3
[TNF-TNFR] + [TRADD] → [TNF-TNFR-TRADD]	[TNF-TNFR] × [TRADD] × k7f
[TNF-TNFR-TRADD] + [TRAF2] + [RIP1] ↔ [TNFR Complex1]	[TNF-TNFR-TRADD] × [TRAF2] × [RIP1] × k8f - k8b × [TNFR Complex1]
[TNFR Complex1] + [FADD] ↔ [TNFR C3]	[TNFR Complex1] × [FADD] × [FADD] × konFADD_R - [TNFR C3] × koff_FADD_r
[XIAP:C3·] → [C3·] + Φ	[XIAP:C3·] × K8
[C8·] + [Bid] ↔ [C8·:Bid]	k10 × [C8*] × [Bid] - [C8·:Bid] × k_10
[tBid] + [BCL-2c] ↔ [tBid:BCL-2c]	k11 × [tBid] × [BCL-2c] - k_11 × [tBid:BCL-2c]
[tBid] + [BAX] ↔ [tBid:BAX]	k12 × [tBid] × [BAX] - k_12 × [tBid:BAX]
[FLIP] + [TNFR C3] ↔ [DISC:FLIP]	k2 × [FLIP] × [TNFR C3] - k_2 × [DISC:FLIP]
[CyCr] ↔ [CyC]	k22 × [CyCr] - k_22 × [CyC]
[CyC] + [APAF] ↔ [APAF:CyC]	k23 × [CyC] × [APAF] - k_23 × [APAF:CyC]
[APAF·] + [C9] ↔ [APOP]	k24 × [APAF·] × [C9] - k_24 × [APOP]
[C3] + [APOP] ↔ [Apop:C3]	k25 × [C3] × [APOP] - [Apop:C3] × k_25
[SMACr] ↔ [SMAC]	k26 × [SMACr] - k_26 × [SMAC]
[APOP] + [XIAP] ↔ [Apop:XIAP]	k27 × [APOP] × [XIAP] - [Apop:XIAP] × k_27
[XIAP] + [SMAC] ↔ [SMAC:XIAP]	k28 × [XIAP] × [SMAC] - [SMAC:XIAP] × k_28
[TNFR C3] + [C8] ↔ [R·C8]	k3 × [TNFR C3] × [C8] - k_3 ×[R·C8]
[C3·] + [C6] ↔ [C3·:C6]	k6 × [C3·] × [C6] - k_6 × [C3·:C6]

[TNF-alpha] + [TNFR] ↔ [TNF-TNFR]	k6f × [TNF-alpha] × [TNFR] / (1 + [DSL] / kiDSL_TNFR1) - [TNF-TNFR] × k6b
[C6·] + [C8] ↔ [C6·:C8]	k7 × [C6·] × [C8] - k_7 × [C6·:C8]
[C3·] + [XIAP] ↔ [XIAP:C3·]	k8 ×[C3*] × [XIAP] - k_8 × [XIAP:C3·]
[C3·] + [PARP] ↔ [PARP:C3·]	k9 × [C3·] × [PARP] - k_9 × [PARP:C3·]

Table S9: Chemical Kinetic Parameters

k1	2.4×10^{-4}	$nM^{-1}S^{-1}$	(Albeck et al. 2008)
k_1	0.001	S^{-1}	(Albeck et al. 2008)
K1	1.0×10^{-5}	S^{-1}	(Albeck et al. 2008)
k2	6.0×10^{-4}	$nM^{-1}S^{-1}$	(Albeck et al. 2008)
k_2	0.001	S^{-1}	(Albeck et al. 2008)
k3	6.0×10^{-4}	$nM^{-1}S^{-1}$	(Albeck et al. 2008)
k4	6.0×10^{-4}	$nM^{-1}S^{-1}$	(Albeck et al. 2008)
k5	6.0×10^{-5}	$nM^{-1}S^{-1}$	(Albeck et al. 2008)
k6	6.0×10^{-4}	$nM^{-1}S^{-1}$	(Albeck et al. 2008)
k7	1.8×10^{-5}	$nM^{-1}S^{-1}$	(Albeck et al. 2008)
k8	0.0012	$nM^{-1}S^{-1}$	(Albeck et al. 2008)
k9	6.0×10^{-5}	$nM^{-1}S^{-1}$	(Albeck et al. 2008)
k10	6.0×10^{-5}	$nM^{-1}S^{-1}$	(Albeck et al. 2008)
k_3	0.001	S^{-1}	(Albeck et al. 2008)
k_4	0.001	S^{-1}	(Albeck et al. 2008)
k_5	0.001	S^{-1}	(Albeck et al. 2008)
k_6	0.001	S^{-1}	(Albeck et al. 2008)
k_7	0.001	S^{-1}	(Albeck et al. 2008)

k_8	0.001	S^{-1}	(Albeck et al. 2008)
k_9	0.01	S^{-1}	(Albeck et al. 2008)
k_10	0.001	S^{-1}	(Albeck et al. 2008)
K3	1	S^{-1}	(Albeck et al. 2008)
K5	1	S^{-1}	(Albeck et al. 2008)
K6	1	S^{-1}	(Albeck et al. 2008)
K7	1	S^{-1}	(Albeck et al. 2008)
K8	0.1	S^{-1}	(Albeck et al. 2008)
K9	1	S^{-1}	(Albeck et al. 2008)
K10	1	S^{-1}	(Albeck et al. 2008)
K12	1	S^{-1}	(Albeck et al. 2008)
K19	1	S^{-1}	(Albeck et al. 2008)
K20	10	S^{-1}	(Albeck et al. 2008)
K21	10	S^{-1}	(Albeck et al. 2008)
K23	1	S^{-1}	(Albeck et al. 2008)
K25	1	S^{-1}	(Albeck et al. 2008)
k11	6.0×10^{-4}	$nM^{-1}S^{-1}$	(Albeck et al. 2008)
k12	6.0×10^{-5}	$nM^{-1}S^{-1}$	(Albeck et al. 2008)
k13	0.01	S^{-1}	(Albeck et al. 2008)
k14	6.0×10^{-4}	$nM^{-1}S^{-1}$	(Albeck et al. 2008)
k15	6.0×10^{-4}	$nM^{-1}S^{-1}$	(Albeck et al. 2008)
k16	6.0×10^{-4}	$nM^{-1}S^{-1}$	(Albeck et al. 2008)
k17	6.0×10^{-4}	$nM^{-1}S^{-1}$	(Albeck et al. 2008)
k18	6.0×10^{-4}	$nM^{-1}S^{-1}$	(Albeck et al. 2008)

k19	6.0×10^{-4}	$nM^{-1}S^{-1}$	(Albeck et al. 2008)
k20	0.0012	$nM^{-1}S^{-1}$	(Albeck et al. 2008)
k_11	0.001	S^{-1}	(Albeck et al. 2008)
k_12	0.001	S^{-1}	(Albeck et al. 2008)
k_13	0.01	S^{-1}	(Albeck et al. 2008)
k_14	0.001	S^{-1}	(Albeck et al. 2008)
k_15	0.001	S^{-1}	(Albeck et al. 2008)
k_16	0.001	S^{-1}	(Albeck et al. 2008)
k_17	0.001	S^{-1}	(Albeck et al. 2008)
k_18	0.001	S^{-1}	(Albeck et al. 2008)
k_19	0.001	S^{-1}	(Albeck et al. 2008)
k_20	0.001	S^{-1}	(Albeck et al. 2008)
k_21	0.001	S^{-1}	(Albeck et al. 2008)
k_22	0.01	S^{-1}	(Albeck et al. 2008)
k_23	0.001	S^{-1}	(Albeck et al. 2008)
k_24	0.001	S^{-1}	(Albeck et al. 2008)
k_25	0.001	S^{-1}	(Albeck et al. 2008)
k_26	0.01	S^{-1}	(Albeck et al. 2008)
k_27	0.001	S^{-1}	(Albeck et al. 2008)
k_28	0.001	S^{-1}	(Albeck et al. 2008)
k21	0.0012	$nM^{-1}S^{-1}$	(Albeck et al. 2008)
k22	0.01	S^{-1}	(Albeck et al. 2008)
k23	3.0×10^{-4}	$nM^{-1}S^{-1}$	(Albeck et al. 2008)
k24	3.0×10^{-5}	$nM^{-1}S^{-1}$	(Albeck et al. 2008)

k25	3.0×10^{-6}	$nM^{-1}S^{-1}$	(Albeck et al. 2008)
k26	0.01	S^{-1}	(Albeck et al. 2008)
k27	0.0012	$nM^{-1}S^{-1}$	(Albeck et al. 2008)
k28	0.0042	$nM^{-1}S^{-1}$	(Albeck et al. 2008)
v	0.07	dimensionless	(Albeck et al. 2008)
k9f	1.21×10^{-7}	$nM^{-2}S^{-1}$	(Schliemann et al. 2011)
kA9d	5.65×10^{-5}	S^{-1}	(Schliemann et al. 2011)
kiDSL_TNFR1	38070	nM	(Bhattacharya, Gachhui, & Sil, 2013)
konFADD_R	1.21×10^{-7}	$nM^{-2}S^{-1}$	(Schliemann et al. 2011)
koff_FADD_r	0.114	S^{-1}	(Schliemann et al. 2011)
k6f	5.38	$\mu M^{-1}S^{-1}$	(Schliemann et al. 2011)
k6b	0.0277	S^{-1}	(Schliemann et al. 2011)
k7f	0.00575	$nM^{-1}S^{-1}$	(Schliemann et al. 2011)
k8b	0.00113	S^{-1}	(Schliemann et al. 2011)
k8f	1.0×10^{-6}	$nM^{-2}S^{-1}$	(Schliemann et al. 2011)
kind_DSL_BCL2	1207	nM	(Bhattacharya, Gachhui, et al., 2013)

2.4. In Silico Modeling of β-glucuronidase Synthesis Pathway in Hepatic Cells

Table S10: Initial Concentrations

Species	Value (nM)	Ref
LPS (Normal)	0.0005	(L. Xue et al., 2017)
LPS (Liver Toxicity)	0.0102	(L. Xue et al. 2017)

IKK(i)	100	(Hoffmann et al., 2002)
IRAK1	0.019	(Gutiérrez, III, & Urcuqui-Inchima, 2010)
IRAK4	0.321	(Gutiérrez, III, & Urcuqui-Inchima, 2010)
MyD88	0.987	(Gutiérrez, III, & Urcuqui-Inchima, 2010)
TABTAK	0.841	(Gutiérrez, III, & Urcuqui-Inchima, 2010)
TLR4	0.111	(Gutiérrez, III, & Urcuqui-Inchima, 2010)
TRAF6	0.668	(Gutiérrez, III, & Urcuqui-Inchima, 2010)
NFkB	100	(Hoffmann et al., 2002)
IKB(a)-NFkB	100.0	(Hoffmann et al., 2002)
MD2	0.54	https://www.genecards.org/cgi-bin/carddisp.pl?gene=LY96&keywords=MD2

Glossary:
LPS - Lipopolysaccharides
IKK(i) - Inhibitor of nuclear factor-κB kinase
IRAK1- Interleukin 1 receptor associated kinase 1
IRAK4 - Interleukin 1 receptor associated kinase 4
MyD88 - Myeloid differentiation primary response protein 88
TABTAK - Transforming growth factor β-activated protein kinase 1 (TAK1)-binding protein
TLR4 - Toll-like receptor 4
TRAF6 - TNF-receptor-associated factor 6
NFkB - Nuclear factor kappa-light-chain-enhancer of activated B cells
IKB(α) - nuclear factor of kappa light polypeptide gene enhancer in B-cells inhibitor, α
MD2 - Myeloid Differentiation factor 2

Table S11: Biochemical Reactions and Rate Equations

Biochemical Reaction	Rate Equation
[LPS_MD2_TLR4] + [MyD88] ↔ [LPS_MD2_TLR4_MyD88]	[LPS_MD2_TLR4]× [MyD88] × kHf1 - [LPS_MD2_TLR4_MyD88]× kHb1 × scale
[LPS_MD2_TLR4_MyD88] + [IRAK4] ↔ [LPS_MD2_TLR4_MYD88_IRAK4]	([LPS_MD2_TLR4_MyD88] × [IRAK4] × kassIRAK4 - [LPS_MD2_TLR4_MYD88_IRAK4] × kdiss_IRAK4) × scale
[LPS_MD2_TLR4_MYD88_IRAK4] → [IRAK4_P]	[LPS_MD2_TLR4_MYD88_IRAK4] × kIRAK4_p × scale
[IRAK4_P] + [IRAK1]↔[IRAK1_IRAK4_p]	([IRAK4_P] × [IRAK1] × kassIRAK4_IRAK1 - [IRAK1_IRAK4_p] × kdissIRAK4_IRAK1) × scale
[IRAK4_P]→[IRAK4]	[IRAK4_P] × kdep_IRAK4 × scale
[IRAK1_IRAK4_p]→[IRAK1_P]+[IRAK4_P]	[IRAK1_IRAK4_p] × kphos_IRAK1 × scale
[IRAK1_P]→[IRAK1]	[IRAK1_P] × kdephos_IRAK1 × scale
[IRAK1_P] + [TRAF6]↔[IRAK1P_TRAF6]	([IRAK1_P] × [TRAF6] × kassIRAK1_TRAF6 - [IRAK1P_TRAF6] × kdissIRAK1_TRAF6) × scale
[TABTAK] + [IRAK1P_TRAF6]↔[IRAK1_TRAF6_TABTAK]	([TABTAK] × [IRAK1P_TRAF6] × kassTBAKTAK - [IRAK1_TRAF6_TABTAK] × kdissTBKTAK) × scale
[IRAK1_TRAF6_TABTAK]→[TABTAK_P] +[IRAK1P_TRAF6]	[IRAK1_TRAF6_TABTAK] × kphos_TBATAK × scale
[TABTAK_P]→ [TABTAK	[TABTAK_P] × kdephos_TABTAK × scale
[TABTAK_P] +[IKK(i)]↔[TABTAK_P_IKK]	[TABTAK_P] × [IKK(i)] × kasIKK_TABTAK × 50 - [TABTAK_P_IKK] × kdissIKK_TABTAK
Φ→ [IKB(a)]	tr1 × [IKB(a) mRNA]
[IKB(a)]→Φ	deg1 × [IKB(a)]
[IKK (a)] + [IKB(a)]↔[IKK-IKB(a)]	[NFkB] × [IKB(a)] × a4 - [IKB(a)-NFkB] × d4
[IKK (a)]+[IKB(a)-NFkB]↔[IKK-IKB(a)-NFkB]	a7 × [IKK (a)] × [IKB(a)-NFkB] - d1 × [IKK-IKB(a)-NFkB]

[NFkB] + [IKB(a)]↔ [IKB(a)-NFkB]	[IKK-IKB(a)-NFkB] × r4 / (1 + DSL / ki_DSL_NFkB)
[NFkB [N]]→[NFkB]	[NFkB [N]] × k01
[IKB(a) mRNA]→Φ	tr3 × [IKB(a) mRNA]
Φ → [IKB(a) mRNA]	tr2a + tr2 × [NFkB [N]] × [NFkB [N]]
[TABTAK_P_IKK]→[IKK (a)] +[TABTAK_P]	kphos_IKK × [TABTAK_P_IKK]
[TLR4] + [MD2] ↔ [TLR4_MD2]	([TLR4] × [MD2] × konMD2_TLR4 - [TLR4_MD2] × koff_MD2_TLR4) × scale
[LPS] + [MD2] ↔ [MD2_LPS]	([LPS] × [MD2] × konMD2_LPS - [MD2_LPS] × koffMD2_LPS) × scale
[TLR4_MD2] + [LPS] ↔ [LPS_MD2_TLR4]	([TLR4_MD2] × [LPS] × konMD2_LPS - [LPS_MD2_TLR4] × koffMD2_LPS) × scale
[TLR4] + [MD2_LPS] ↔ [LPS_MD2_TLR4]	([TLR4] × [MD2_LPS] × konMD2_TLR4 - [LPS_MD2_TLR4] × koff_MD2_TLR4) × scale
[LPS] + [TLR4] ↔ [TLR4_LPS]	([LPS] × [TLR4] × kon_TLR4_LPS - [TLR4_LPS] × koff_TLR4_LPS) × scale
[TLR4_LPS] +[MD2] ↔ [LPS_MD2_TLR4]	[TLR4_LPS] × [MD2] × konMD2_TLR4 - [LPS_MD2_TLR4] × koff_MD2_TLR4) × scale
Φ → [b-Glucuronidase mRNA]	[NFkB [N]] × ksyn_bglucmRNA
[b-Glucuronidase mRNA] → [b-glucuronidase mRNA-c]	[b-Glucuronidase mRNA] × ktrans
Φ ↔ [b-Glucuronidase]	[b-glucuronidase mRNA-c] × ksyn_bgluc × scale1 - [b-Glucuronidase] × kdeg_bgludase × scale1
[b-glucuronidase mRNA-c] → Φ	[b-glucuronidase mRNA-c] × kdeg_bgludasemrNA × scale1
[IKK(i)] → [IKK (a)]	[IKK(i)] × [ROS] × 1e-05 × k10f / (k10m + [IKK(i)]) - [IKK (a)] × k10b
[IKK (a)] → Φ	k02 × [IKK (a)]

Table S12: Chemical Kinetic Parameters

Parameter	Value	Units	Reference
a1	2.25×10^{-5}	$nM^{-1}s^{-1}$	(Hoffmann et al., 2002)
a4	5.0×10^{-4}	$nM^{-1}s^{-1}$	(Hoffmann et al., 2002)
a6	5.0×10^{-4}	$nM^{-1}s^{-1}$	(Hoffmann et al., 2002)
a7	1.85×10^{-4}	$nM^{-1}s^{-1}$	(Hoffmann et al., 2002)
a8	4.8×10^{-5}	$nM^{-1}s^{-1}$	(Hoffmann et al., 2002)
a9	7.0×10^{-5}	$nM^{-1}s^{-1}$	(Hoffmann et al., 2002)
d1	0.00125	S^{-1}	(Hoffmann et al., 2002)
d4	5.0×10^{-4}	S^{-1}	(Hoffmann et al., 2002)
d6	5.0×10^{-4}	S^{-1}	(Hoffmann et al., 2002)
r1	0.00407	S^{-1}	(Hoffmann et al., 2002)
r4	0.0204	S^{-1}	(Hoffmann et al., 2002)
r6	0.011	S^{-1}	(Hoffmann et al., 2002)
deg1	1.0×10^{-4}	S^{-1}	(Hoffmann et al., 2002)
tr2a	0.0015	nMs^{-1}	(Hoffmann et al., 2002)
tr1	0.004	S^{-1}	(Hoffmann et al., 2002)
tr2	1.7×10^{-5}	$nM^{-1}s^{-1}$	(Hoffmann et al., 2002)
tr3	2.8×10^{-4}	S^{-1}	(Hoffmann et al., 2002)
tp1	3.0×10^{-4}	S^{-1}	(Hoffmann et al., 2002)
tp2	2.0×10^{-4}	S^{-1}	(Hoffmann et al., 2002)
k02	1.2×10^{-4}	S^{-1}	(Hoffmann et al., 2002)
kHf1	0.0995	$nM^{-1}s^{-1}$	(Gutiérrez, III, & Urcuqui-Inchima, 2010)
kHb1	0.0988	S^{-1}	(Gutiérrez, III, & Urcuqui-Inchima, 2010)
kassHMGB1_TLR4	1.54×10^{-6}	$nM^{-1}s^{-1}$	(Yang et al. 2010)
kdissHMGB1_TLR4	0.00231	S^{-1}	Calculated
kassIRAK4	0.098	$nM^{-1}s^{-1}$	(Gutiérrez, III, & Urcuqui-Inchima, 2010)

kdiss_IRAK4	0.0994	S^{-1}	(Gutiérrez, III, & Urcuqui-Inchima, 2010)
kIRAK4_p	1.0×10^{-4}	S^{-1}	(Gutiérrez, III, & Urcuqui-Inchima, 2010)
kdep_IRAK4	0.0038	S^{-1}	(Gutiérrez, III, & Urcuqui-Inchima, 2010)
kassIRAK4_IRAK1	5.9×10^{-5}	$nM^{-1}s^{-1}$	(Gutiérrez, III, & Urcuqui-Inchima, 2010)
kdissIRAK4_IRAK1	0.0023	S^{-1}	(Gutiérrez, III, & Urcuqui-Inchima, 2010)
kphos_IRAK1	1.8×10^{-4}	S^{-1}	(Gutiérrez, III, & Urcuqui-Inchima, 2010)
kdephos_IRAK1	0.0012	S^{-1}	(Gutiérrez, III, & Urcuqui-Inchima, 2010)
kassIRAK1_TRAF6	0.002	$nM^{-1}s^{-1}$	(Gutiérrez, III, & Urcuqui-Inchima, 2010)
kdissIRAK1_TRAF6	4.9×10^{-4}	S^{-1}	(Gutiérrez, III, & Urcuqui-Inchima, 2010)
kassTBAKTAK	3.3×10^{-4}	$nM^{-1}s^{-1}$	(Gutiérrez, III, & Urcuqui-Inchima, 2010)
kdissTBKTAK	1.8×10^{-4}	S^{-1}	(Gutiérrez, III, & Urcuqui-Inchima, 2010)
kphos_TBATAK	0.0019	S^{-1}	(Gutiérrez, III, & Urcuqui-Inchima, 2010)
kdephos_TABTAK	3.7×10^{-4}	S^{-1}	(Gutiérrez, III, & Urcuqui-Inchima, 2010)
kasIKK_TABTAK	1.2×10^{-4}	$nM^{-1}s^{-1}$	(Gutiérrez, III, & Urcuqui-Inchima, 2010)
kdissIKK_TABTAK	1.0×10^{-4}	S^{-1}	(Gutiérrez, III, & Urcuqui-Inchima, 2010)
kphos_IKK	8.8×10^{-4}	S^{-1}	(Gutiérrez, III, & Urcuqui-Inchima, 2010)
kdephosIKK	0.0014	S^{-1}	(Gutiérrez, III, & Urcuqui-Inchima, 2010)
ksynIL1	0.0037641	nMs^{-1}	(Yoza et al. 1998)
kdegIL1mRNA	2.3×10^{-4}	S^{-1}	(Yoza et al. 1998)

kdegIL1	1.283×10^{-5}	S^{-1}	(Ainscough et al. 2014)
ksynIL1mRNA	0.00232	S^{-1}	(Arsdell et al. 2000)
ktrans	0.005775	S^{-1}	Fixed using ref (Dargemont & Kuhn, 1992)
kon_TLR4_LPS	3.23×10^{-4}	$nM^{-1}s^{-1}$	(Shin et al., 2007)
koff_TLR4_LPS	0.0454	S^{-1}	(Shin et al. 2007)
konMD2_LPS	5.61×10^{-4}	$nM^{-1}s^{-1}$	(Shin et al. 2007)
koffMD2_LPS	0.0128	S^{-1}	(Shin et al. 2007)
konMD2_TLR4	7.5×10^{-4}	$nM^{-1}s^{-1}$	(Shin et al. 2007)
koff_MD2_TLR4	4.7×10^{-4}	S^{-1}	(Shin et al. 2007)
kdeg_bgludase mrNA	8.021×10^{-6}	S^{-1}	(Watson, Daveyl, Labarca, & Paigen, 1981)
kdeg_bgludase	8.021×10^{-6}	S^{-1}	(Watson et al. 1981)
ksyn_bglucmRNA	0.591	$nM\ S^{-1}$	(Bracey & Paigen, 1987)
ksyn_bgluc	1.91×10^{-5}	$nM\ S^{-1}$	(Bracey and Paigen 1987)
ki_DSL_NFkB	57106	nM	(Bhattacharya, Manna, et al., 2013)
k10f	0.1	S^{-1}	(Sharp, Ma, Saunders, & Norman, 2013)
k10b	0.1	S^{-1}	(Sharp et al. 2013)
k10m	1×10^{5}	nM	(Sharp et al. 2013)

1. Ainscough, Joseph S., G. Frank Gerberick, Maryam Zahedi-Nejad, Gloria Lopez-Castejon, David Brough, Ian Kimber, and Rebecca J. Dearman. 2014. "Dendritic Cell IL-1?? And IL-1?? Are Polyubiquitinated and Degraded by the Proteasome." *Journal of Biological Chemistry* 289 (51): 35582–92. doi:10.1074/jbc.M114.595686.
2. Aitken, R. J., & Roman, S. D. (2008). Antioxidant systems and oxidative stress in the testes (Reprinted from Molecular Mechanisms in Spermatogenesis, 2007). *Oxidative Medicine and Cellular Longevity*, *1*(1), 15–24.
3. Albeck, J. G., Burke, J. M., Spencer, S. L., Lauffenburger, D. A., & Sorger, P. K. (2008). Modeling a Snap-Action , Variable-Delay Switch Controlling Extrinsic Cell Death. *PLoS Biology*, *6*(12), 2831–2852. http://doi.org/10.1371/journal.pbio.0060299
4. Ambrosio, G., Zweier, J. A. Y. L., Jacobus, W. E., Ph, D., Weisfeldt, M. L., & D, J. T. F. M. (1987). Improvement of postischemic myocardial function and metabolism induced by administration of deferoxamine at the time of reflow : the role of iron in the pathogenesis of reperfusion injury O02. *Circulation*, *76*(4), 1987.
5. Arsdell, S. W. Van, Murphy, K. P., Pazmany, C., Erickson, D., Burns, C., & Moody, M. D. (2000). Xplore ® mRNA Assays for the Quantification of IL-1 β and TNF- α mRNA in Lipopolysaccharide- Induced Mouse Macrophages. *BioTechniques*, *28*(6), 1–4.
6. Atunes, F., Salvador, A., Marinho, H. S., Alves, R., & Pinto, R. E. (1996). Lipid Peroxidation in Mitochondrial inner membranes. An integrative kinetic model. *Free Radical Biology and Medicine*, *21*(7), 917–943.
7. Aw, T. Y., & Jones, D. P. (1982). Direct determination of UDP-glucuronic acid in cell extracts by high-performance liquid chromatography. *Analytical Biochemistry*, *127*(1), 32–36. http://doi.org/10.1016/0003-2697(82)90140-3
8. Aydemir, T., & Kuru, K. (2003). Purification and Partial Characterization of Catalase from Chicken Erythrocytes and the Effect of Various Inhibitors on Enzyme Activity. *Turk.J.Chem*, *27*, 85–97.
9. Babbs, C. F., & Steiner, M. G. (1990). Simulation of free radical reactions in biology and medicine: A new two-compartment kinetic model of intracellular lipid peroxidation. *Free Radical Biology and Medicine*, *8*, 471–485.
10. Bhattacharya, S., Chatterjee, S., Manna, P., Das, J., Ghosh, J., Gachhui, R., & Sil, P. C. (2011). Prophylactic role of D-saccharic acid-1,4-lactone in tertiary butyl hydroperoxide induced cytotoxicity and cell death of murine hepatocytes via mitochondria-dependent pathways. *Journal of Biochemical and Molecular Toxicology*, *25*(6), 341–354. http://doi.org/10.1002/jbt.20393

11. Bhattacharya, S., Gachhui, R., & Sil, P. C. (2013). The prophylactic role of D-saccharic acid-1,4-lactone against hyperglycemia-induced hepatic apoptosis via inhibition of both extrinsic and intrinsic pathways in diabetic rats. *Food & Function*, *4*, 283–296. http://doi.org/10.1039/c2fo30145h

12. Bhattacharya, S., Manna, P., Gachhui, R., Sil, P. C., Sciences, L., & Road, R. S. C. M. (2013). D-Saccharic acid 1 , 4-lactone protects diabetic rat kidney by ameliorating hyperglycemia-mediated oxidative stress and renal inflammatory cytokines via NF-κ B and PKC signaling. *Toxicology and Applied Pharmacology*, *267*(1), 16–29. http://doi.org/10.1016/j.taap.2012.12.005

13. Boase, S., & Miners, J. O. (2002). In vitro – in vivo correlations for drugs eliminated by glucuronidation : Investigations with the model substrate zidovudine, *450*, 493–503.

14. Bracey, L. T., & Paigen, K. (1987). Changes in translational yield regulate tissue-specific expression of β-glucuronidase. *Proc.Natl.Acad.Sci.USA*, *84*(December), 9020–9024.

15. Buettner, G. R., Ng, C. F., Wang, M., Rodgers, V. G. J., & Schafer, F. Q. (2006). A new paradigm: manganese superoxide dismutase influences the production of H2O2 in cells and thereby their biological state. *Free Radical Biology & Medicine*, *41*(8), 1338–50. http://doi.org/10.1016/j.freeradbiomed.2006.07.015

16. Dargemont, C., & Kuhn, L. C. (1992). Export of mRNA from Microinjected Nuclei of Xenopus laevis Oocytes. *The Journal of Cell Biology*, *118*(1), 1–9.

17. Dwivedi, C., Heck, W. J., Downie, A. A., Larroya, S., & Webb, T. E. (1990). Effect of calcium glucarate on β-glucuronidase activity and glucarate content of certain vegetables and fruits. *Biochemical Medicine and Metabolic Biology*, *43*(2), 83–92. http://doi.org/10.1016/0885-4505(90)90012-P

18. Edwards, A., Cao, C., & Pallone, T. L. (2011). Cellular mechanisms underlying nitric oxide-induced vasodilation of descending vasa recta. *American Journal of Physiology. Renal Physiology*, *300*(2), F441-56. http://doi.org/10.1152/ajprenal.00499.2010

19. Feng S, Song JD. Determination of β-glucuronidase in human colorectal carcinoma cell lines (1997). *World J Gastroenterol*. *3(4)*, 251-252. doi: 10.3748/wjg.v3.i4.251.

20. Gutiérrez, Jayson, Georges St Laurent, and Silvio Urcuqui-Inchima. (2010). "Propagation of Kinetic Uncertainties through a Canonical Topology of the TLR4 Signaling Network in Different Regions of Biochemical Reaction Space." *Theoretical Biology & Medical Modelling* 7 (1): 7. doi:10.1186/1742-4682-7-7.

21. H.Stillinger, F. (1978). Proton Transfer Reactions and Kinetics in Water. In *Theoretical Chemistry, Advances and Perspedives* (pp. 178–234).

22. Hanausek, M., Walaszek, Z., & Slaga, T. J. (2003). Detoxifying Cancer Causing Agents to Prevent Cancer, *2*(2), 139–144. http://doi.org/10.1177/1534735403253305

23. Harte, A. L., Silva, N. F., Creely, S. J., Mcgee, K. C., Billyard, T., Youssef-elabd, E. M., … Mcternan, P. G. (2010). Elevated endotoxin levels in non-alcoholic fatty liver disease. *Journal of Inflammation*, *7*(15), 1–10.

24. Henle, E.S., Luo, Y., and Linn, S. (1996). Fe2+ , Fe 3+, and Oxygen React with DNA-Derived Radicals Formed during iron mediated Fenton reactions. Biochemistry 35, 12212–12219.

25. Hoffmann, A., Levchenko, A., Scott, M.L., and Baltimore, D. (2002). The IkappaB-NF-kappaB signaling module: temporal control and selective gene activation. Science 298, 1241–1245.

26. Kavida, M. (2011). Mathematical and Computational Models of Oxidative and Nitrosative Stress. *Crit Rev Biomed Eng.*, *39*(5), 461–472. http://doi.org/10.1002/cphy.c100054.REACTIVE

27. Lampe, J. W., Li, S. S., Potter, J. D., & King, I. B. (2002). Serum Beta-Glucuronidase Activity Is Inversely Associated with Plant Food intakes in humans. *Cancer*, *2*(January), 1341–1344.

28. Li, C. P., Li, J. H., He, S. Y., Li, P., & Zhong, X. L. (2014). Roles of Fas/Fasl, Bcl-2/Bax, and Caspase-8 in rat nonalcoholic fatty liver disease pathogenesis. *Genetics and Molecular Research*, *13*(2), 3991–3999. http://doi.org/10.4238/2014.May.23.10

29. Li, J., Ke, W., Zhou, Q., Wu, Y., Luo, H., Zhou, H., … Guo, Y. (2014). Tumour necrosis factor- a promotes liver ischaemia-reperfusion injury through the PGC-1 a / Mfn2 pathway. *J.Cell.Mol.Med.*, *18*(9), 1863–1873. http://doi.org/10.1111/jcmm.12320

30. Loegering, D. J., & Lennartz, M. R. (2011). Protein kinase C and toll-like receptor signaling. *Enzyme Research*, *2011*(1). http://doi.org/10.4061/2011/537821

31. Macfarlane, N. G., & Miller, D. J. (1992). Depression of Peak Force Without Altering Calcium Sensitivit by the Superoxide Anion in Chemically Skinned Cardiac Muscle of Rat. *Circulation Research*, *70*(6), 1217–1224.

32. Mandrekar, P., & Ambade, A. (2012). Cellular Signaling Pathways in Alcoholic Liver Disease. In *Trends in Alcoholic Liver Disease Research - Clinical and Scientific Aspects* (Vol. 2, p. 64). InTech. http://doi.org/10.5772/27412

33. Nichols, J. W., Ladd, M. A., Fitzsimmons, P. N., & Al, N. E. T. (2017). Measurement of Kinetic Parameters for Biotransformation of Polycyclic Aromatic Hydrocarbons by Trout

Liver S9 Fractions : Implications for Bioaccumulation Assessment, *XX*(Xx), 1–14. http://doi.org/10.1089/aivt.2017.0005

34. Nolan, J. P., Hare, D. K., McDevitt, J. J., & Vilayat Ali, M. (1977). In Vitro Studies of Intestinal Endotoxin Absorption: I. Kinetics of absorption in the isolated everted gut sac. *Gastroenterology*, *72*(3), 434–439. http://doi.org/10.1016/S0016-5085(77)80253-9

35. Peyrol, J., Meyer, G., Obert, P., Dangles, O., Pechère, L., Amiot, M. J., & Riva, C. (2018). Involvement of bilitranslocase and beta-glucuronidase in the vascular protection by hydroxytyrosol and its glucuronide metabolites in oxidative stress conditions. *Journal of Nutritional Biochemistry*, *51*, 8–15. http://doi.org/10.1016/j.jnutbio.2017.09.009

36. Rampal, G., Khanna, N., Thind, T. S., Arora, S., & Vig, A. P. (2012). Role of isothiocyanates as anticancer agents and their contributing molecular and cellular mechanisms. *Medicinal Chemistry & Drug Discovery*, *3*(2), 79–93.

37. Schliemann, M., Bullinger, E., Borchers, S., Allgöwer, F., Findeisen, R., & Scheurich, P. (2011). Heterogeneity reduces sensitivity of cell death for TNF-Stimuli. *BMC Systems Biology*, *5*(1), 204. http://doi.org/10.1186/1752-0509-5-204

38. Sharp, G. C., Ma, H., Saunders, P. T. K., & Norman, J. E. (2013). A Computational Model of Lipopolysaccharide-Induced Nuclear Factor Kappa B Activation : A Key Signalling Pathway in Infection-Induced Preterm Labour. *PLOS One*, *8*(7), 1–6. http://doi.org/10.1371/journal.pone.0070180

39. Shin, H. J., Lee, H., Park, J. D., Hyun, H. C., Sohn, H. O., Lee, D. W., & Kim, Y. S. (2007). Molecules and Kinetics of Binding of LPS to Recombinant CD14 , TLR4 , and MD-2 Proteins. *Molecules and Cells*, *24*(1), 119–124.

40. Subramanian, G., & Madras, G. (2016). Introducing saccharic acid as an ef fi cient iron chelate to enhance photo-Fenton degradation of organic contaminants. *Water Research*, *104*, 168–177.

41. Taylor, D. J., Styles, P., Matthews, P. M., & Gadian, D. G. (1986). Energetics of Human Muscle : Exercise-Induced ATP Depletion. *Magnetic Resonance in Medicine*, *3*, 44–54.

42. Tomasic, J., & Keglevic, D. (1973). The kinetics of hydrolysis of synthetic glucuronic esters and glucuronic ethers by bovine liver and Escherichia coli b-glucuronidase. *Biochemical Journal*, *133*, 789–795. http://doi.org/10.1042/bj1330789

43. Wang, Y., Branicky, R., Noë, A., & Hekimi, S. (2018). Superoxide dismutases: Dual roles in controlling ROS damage and regulating ROS signaling. *Journal of Cell Biology*, *217*(6), 1915–1928. http://doi.org/10.1083/jcb.201708007

44. Watson, G., Daveyl, R. A., Labarca, C., & Paigen, K. (1981). Genetic determination of kinetic parameters in β-glucuronidase induction by androgen. *The Journal of Biologcal Chemistry*, *256*(6), 3005–3011.

45. Xue, C., Chou, C.-S., Kao, C.-Y., Sen, C. K., & Friedman, A. (2012). Propagation of Cutaneous Thermal Injury: A Mathematical Model. *Wound Repair Regeneration*, *20*(1), 114–122. http://doi.org/10.1111/j.1524-475X.2011.00759.x

46. Xue, L., He, J., Gao, N., Lu, X., Li, M., Wu, X., … Jin, Y. (2017). Probiotics may delay the progression of nonalcoholic fatty liver disease by restoring the gut microbiota structure and improving intestinal endotoxemia. *Nature Publishing Group*, *7*, 1–13. http://doi.org/10.1038/srep45176

47. Yang, Huan, Hulda S Hreggvidsdottir, Karin Palmblad, Haichao Wang, Mahendar Ochani, Jianhua Li, Ben Lu, et al. 2010. "A Critical Cysteine Is Required for HMGB1 Binding to Toll-like Receptor 4 and Activation of Macrophage Cytokine Release." *Proceedings of the National Academy of Sciences of the United States of America* 107 (26): 11942–47. doi:10.1073/pnas.1003893107.

48. Yao, D., Dong, Q., Tian, Y., Dai, C., & Wu, S. (2018). Lipopolysaccharide stimulates endogenous β-glucuronidase via PKC/NF-κB/c-myc signaling cascade: a possible factor in hepatolithiasis formation. *Molecular and Cellular Biochemistry*, *444*(1–2), 93–102. http://doi.org/10.1007/s11010-017-3234-3

49. Yoza, Barbara K, Jon D Wells, Charles E Mccall, J O N D Wells, and Charles E M C Call. 1998. "Interleukin-1 β Expression after Inhibition of Protein Phosphatases in Endotoxin-Tolerant Cells." *Clinical and Vaccine Immunology* 5 (3): 281–87.

Supplementary File S3

Medical Subject Heading (MeSH) Keywords

The specific list of Medical Subject Headings (MeSH) keywords is provided below. The literature review was restricted to articles published during the time period of January 1980 to March 2019.

Table S13: Search strings used to identify literature.

**Keywords**
1. Signaling Pathways inducing Liver toxicity
2. Reactive oxygen species AND liver toxicity
3. Glucuronidation signaling Pathway
4. Glucaric acid AND Liver detoxification
5. Glucaric acid AND Oxidative stress
6. Glucaric acid AND β-glucuronidase
7. Glucaric acid AND hepatic apoptosis
8. β-glucuronidase AND Liver toxicity
9. Apoptosis AND liver toxicity
